# The “Parallel Pandemic” in the Context of China: The Spread of Rumors and Rumor-Corrections During COVID-19 in Chinese Social Media

**DOI:** 10.1177/00027642211003153

**Published:** 2021-12

**Authors:** Yunya Song, K. Hazel Kwon, Yin Lu, Yining Fan, Baiqi Li

**Affiliations:** 1 Hong Kong Baptist University, Kowloon, Hong Kong; 2 Arizona State University, Phoenix, AZ, USA; 3 Independent scholar, Brookhaven, GA, USA

**Keywords:** COVID-19, rumor, rumor-correction, frames, emotion, rationality, source characteristics

## Abstract

Although studies have investigated cyber-rumoring previous to the pandemic, little research has been undertaken to study rumors and rumor-corrections during the COVID-19 (coronavirus disease 2019) pandemic. Drawing on prior studies about how online stories become viral, this study will fill that gap by investigating the retransmission of COVID-19 rumors and corrective messages on Sina Weibo, the largest and most popular microblogging site in China. This study examines the impact of rumor types, content attributes (including frames, emotion, and rationality), and source characteristics (including follower size and source identity) to show how they affect the likelihood of a COVID-19 rumor and its correction being shared. By exploring the retransmission of rumors and their corrections in Chinese social media, this study will not only advance scholarly understanding but also reveal how corrective messages can be crafted to debunk cyber-rumors in particular cultural contexts.

As of January 19, 2021, the COVID-19 (coronavirus disease 2019) outbreak has affected the lives of global citizens across 221 countries (Worldometer, 2021). In many networked societies around the world, the COVID-19 information landscape has centered on social media. China—the country where the outbreak began in late December 2019—has been no exception. Social media have blended a range of sources including mainstream news, government and nongovernment organizations, citizen journalists, and laymen’s personal thoughts and stories (Papacharissi, 2015). While much shared information on social media has been well-intended and reliable, false information has likewise flourished. As the pandemic spreads globally, so too does a parallel pandemic of misinformation.

Rumors and unverified claims come in many forms, from conspiracy theories about the virus being created as a biological weapon in China to claims that cats and dogs can spread the coronavirus. At its worst, some health-related rumors have introduced people to ineffective or even potentially harmful remedies, causing them to either overreact (e.g., by hoarding goods) or, more dangerously, underreact (e.g., by engaging in risky behaviors and inadvertently spreading the virus). Chinese officials and social media companies claim to have made substantial efforts to correct widespread false information and recalibrate public discourse about COVID-19. In the early days of the crisis, Chinese authorities tightened social media censorship to curb alerts to the public on the threat of the then-unknown virus (Ruan et al., 2019). As the pandemic developed, social media platforms worked to identify and vet rumors through government-backed entities supervised by the Cyberspace Administration of China (CAC).

Little research has been undertaken to study the psychology of rumors and rumor-correcting in the context of China. Existing studies of rumors in China have mostly focused on rumor detection on social media with automated algorithms (e.g., Lv et al., 2020; Z. Wang & Guo, 2020). Nevertheless, different socioeconomic, political, and media environments create disparate levels of susceptibility to rumors and misinformation (Kwon et al., 2016; Kwon & Rao, 2017; Oh et al., 2018; X. Wang & Song, 2020). To enrich the scholarly understanding of rumor dissemination and debunking under distinct structural conditions, we set out to investigate the retransmission of COVID-19 rumors and corrective messages on Sina Weibo, the largest and most popular microblogging site in China, as an important case study.

On social media, the community of users collectively contribute to disseminating rumors and rumor-correction messages. While research has suggested that online communities have the capacity of self-correction and self-policing, rumor-correction messages have been found at times to be ineffective and even to intensify misperceptions (Goh et al., 2017). Drawing from literature on social sharing of news contents (Berger & Milkman, 2012; Stieglitz & Dang-Xuan, 2013), we examine the impact of rumor types, frames, and source characteristics on sharing rumor and corrective messages.

## Rumors and Rumor-Corrections in the Context of China

Rumors are “claims of fact that have not been shown to be true, but that move from one person to another, and hence have credibility not because direct evidence is known to support them, but because other people seem to believe them” (Sunstein, 2014, p. 6).

As acts of crisis communication, rumors are important sources of information filling in for a dearth of information in a situation where any information could be of value (DiFonzo & Bordia, 1997; Oh et al., 2010). Rumors, however, are “improvised” with unknown veracity (Shibutani, 1966), bypassing the gatekeeping of authorized outlets and mainstream media. Their viral propagation can engender misunderstanding and strike panic (Y. Zhang et al., 2019), especially when the rumor later turns out to be false.

Social media companies have worked in tandem with third-party fact-checkers (as in the cases of Facebook and Twitter) or official government agents (as in the case of Sina Weibo) to respond to rumors and circulate counterpart correction messages (Shin et al., 2017); and such effort has been rightly more aggressive during the pandemic (Brennen et al., 2020). As an integral part of the lifecycle of rumors, rumor-corrections are commonly conceptualized as control strategies that aim to deliberately confine or stop the spread of rumors (Kimmel, 2004; Tripathy et al., 2010). Compared with rumors, rumor-corrections exhibit lower levels of emotion, higher clarity, higher credible source attribution (Chua & Banerjee, 2018), more original content, and fewer URLs (Starbird et al., 2014).

In China, the Chinese government has collaborated with major digital companies and news media outlets in their efforts countering online rumors. For example, the launch of the Chinese Internet Joint Rumor Refutation Platform (the Platform) in 2018 marked extensive collaboration between government institutions and social media giants on what they deemed to be rumor identification, correction, and control in virtual space (Shi, 2018). Social networking sites have been a major battlefield in these collaborative endeavors. In addition, CAC has recommended several fact-checking tools to individual users to verify vetted information on their own, such as the “Verification” section in Xinhua News Agency’s mobile app and the “Fact Check” platform of Tencent News (CAC, 2020).

Sina Weibo sends out a daily digest of popular COVID-19 rumors that government agencies, state-sponsored media, and other authorities determine to be false. Weibo also runs a few specialized rumor-correction entities, such as *Weibo Piyao* (Weibo Rumor Rebuttal) and *Zhuoyaoji* (Rumor Hunt). To understand these collaborative efforts, it is helpful to understand how “false information” (bushi xinxi) has been officially defined in the context of China. Weibo has used the term *false information* [bushi xinxi] to designate posts that contain what is called “factual falsity” and have “social impact” (Sina Weibo, 2020). This two-part definition comprises not only the dimension of factual incorrectness but also certain degrees of social impact. According to the official statistics in a report released by Weibo Piyao (2020) near the time of data collection, there were between 2,000 and 5,000 reported cases of false information as defined above on Weibo each day in May 2020, among which 6,467 cases were removed before the public could see them and 138 rumors that were spread were officially debunked.

## Characterizing Rumors and Correction Messages

Rosnow and Fine (1976) point out that “disasters and other crises are characterized by high importance, high ambiguity, low critical sensibility, and many rumors” (p. 52). As an exemplification, the COVID-19 pandemic has triggered high levels of rumor mongering throughout global digital networks. Some COVID-19 cyberspace rumors have fortunately been debunked, with debunking messages successfully disseminated via social media. That said, many rumors have lingered in social media spaces without receiving a fact-check. Furthermore, a Twitter study (Singh et al., 2020) showed that even when credible information was released, sharing legitimate messages occurred less frequently than sharing misinformation.

The informational asymmetry between rumors and correction messages is not unique to the COVID-19 pandemic. It has been a problem across crisis contexts and political rumors (e.g., Shin et al., 2017; Starbird et al., 2014), calling for better understanding on what makes rumors and correction messages prone to transmissions in social media, and how to strategize in order to better disseminate the correction messages. Drawing on social virality research in both rumoring and nonrumoring contexts, we examine several factors that may affect the transmission of rumors and correction messages.

### Types of Rumors

Knapp (1944) introduced a taxonomy of three types of rumor: (1) “pipe-dream” rumors: rumors that lead to wishful thinking, (2) “bogy” rumors: dread rumors that increase anxiety or fear, and (3) “wedge-driving” rumors: those that generate hatred. In a recent study of Twitter, Chua, Aricat, and Goh (2017) discovered that wish, dread, and wedge-driving are distinguished not only by their underlying emotional appeal but also by their content categories, such as whether the rumor is information-related or of deliberation. Nonetheless, which type of rumor is more likely to go viral and how rumor type affects the sharing of rumors and rumor-corrections in situations have received limited attention.

Among wishful thinking, dread, and wedge-driving rumors, wedge-driving ones have been particularly prominent in times of crisis. Wedge-driving rumors tend to spread easily under a crisis motivated by self-defense (Knapp, 1944). That is, crisis poses a threat to individuals, and threatened individuals act on their anxiety by denigrating others (DiFonzo & Bordia, 2007). Kwon et al. (2016) investigated social media rumors in the context of national security threats in South Korea, finding that wedge-driving rumor narratives were “markedly propagandistic as well as reminiscent of the Cold War era,” which unveiled political hostility toward opposing ideological groups (p. 215).

We hypothesize that different types of rumors should disproportionately influence susceptibility to the original rumor, as well as the perception of the usefulness of its correction message (Dibble, 2014; Willemsen et al., 2011), which in turn increases sharing activities online. Given that the literature has noted the prominence of wedge-driving rumors in times of crisis, we propose the following hypotheses:

**Hypothesis 1:** (a) Wedge-driving rumors about COVID-19 and (b) their correction messages will be shared more frequently than other types of rumors.

### Rumor Framing

Another aspect of message characteristics concerns the ways in which rumor and rumor-correction posts are framed on Weibo. Framing refers to the selection of certain aspects of reality and making them more salient so as to promote a desired interpretation (Entman, 1993). Narrowly defined, media frames refer to the sense-making and interpretive packages used to contextualize events by manipulating metaphors, catchphrases, and images to prioritize some aspects of the event over others (Gamson & Modigliani, 1989; Pippa et al., 2003). Media frames in a public health crisis matter because the emphasis and omission of aspects of the crisis can aggravate or undermine anxiety and panic in the public’s mind (Cohen, 2002; Garland, 2008). As “improvised news” (Shibutani, 1966), rumor messages tend to imitate the breaking news style and thus may employ familiar media frame patterns that often appear in news coverage.

Media frames are generally classified into two categories: generic and issue-specific frames. Generic frames are structural themes including conflict, human interest, economic impact, responsibility, and morality (Semetko & Valkenburg, 2000), while issue-specific frames vary from case to case, depending on the content, topic, and context of study (De Vreese, 2005). Some studies have examined the adoption of responsibility, morality, conflict, and human interest frames in crisis situations (e.g., Gadekar et al., 2014). The attribution of responsibility frames has predominated crisis news coverage (An & Gower, 2009; Bier et al., 2018; Kwon et al., 2019). Given these theoretical concerns, this study posits the following hypotheses:

**Hypothesis 2:** The presence of a (a) responsibility, (b) morality, (c) conflict, or (d) human interest frame in a rumor post increases the likelihood of sharing the rumor post in Weibo.
**Hypothesis 3:** The presence of a (a) responsibility, (b) morality, (c) conflict, or (d) human interest frame in a rumor-correction post increases the likelihood of sharing the correction post in Weibo.

### Emotion and Rationality

Prior research has confirmed emotion and rationality as twin engines of persuasion and responsiveness in online communication (X. Liu et al., 2019; Sahly et al., 2019). Emotional appeals are arousal-inducing features, which can increase social transmission of information (Berger, 2011). Research on how emotion shapes virality has found that contents with sentiment valence, whether positive or negative, are more likely to be shared than those without (Huffaker, 2010; Song et al., 2016; Song et al., 2020).

Meanwhile, rational appeals involve facts and logical statements (Holmes & Crocker, 1987). An argument with higher facticity is more likely to be perceived as trustworthy (Ziegele et al., 2014). Choi’s (2014) study of political discussion on Twitter suggests that cognitive elements, assertion in particular, significantly increase the number of shares a message receives. Using words of certainty, such as “always” and “never,” exhibits a message poster’s confidence in his or her thoughts and raises his or her persuasiveness (Huffaker, 2010).

The spread of rumors during social crisis, according to Allport and Postman (1947), is motivated by “intellectual pressure along with the emotional” (p. 37). Empirical evidence has shown that rumor transmission can be both emotionally and factually driven (Goh et al., 2017; F. Liu et al., 2014). Comparatively, rumor-corrections can be more fact-based (Chua & Banerjee, 2018; Goh et al., 2017). Emotions, however, can also be transmitted from one person to another during the rumor refutation process, and they can affect the effectiveness of rumor-corrections (Zeng & Zhu, 2019). Therefore, we propose the following hypotheses:

**Hypothesis 4:** The presence of (a) emotional or (b) rational appeals in a rumor post increases the likelihood of sharing the rumor post in Weibo.
**Hypothesis 5:** The presence of (a) emotional or (b) rational appeals in a rumor-correction post increases the likelihood of sharing the correction post in Weibo.

### Source Characteristics

Trustworthiness of rumored information heavily relies on source credibility, both in off-line word-of-mouth (Pezzo & Beckstead, 2006; Rosnow, 1991) and cyber-rumoring contexts (Garrett, 2011; Oh et al., 2013). The concept of source dates to Aristotle’s notion of ethos, which refers to an authoritative characteristic of communicators. Messages from an authoritative speaker are more persuasive, exerting greater influence on receivers’ attitudinal changes (Ohanian, 1990) and their judgments of whether to pass along the message or not (Andrews et al., 2016).

Source credibility has been looked on as an important factor in the spread of correction messages in networked environments. For example, Chua and Banerjee (2018) reported that compared with rumors, rumor-corrections were much more likely to hyperlink to credible sources. Also, formal or credible sources have enhanced the spread of health messages in other health communication contexts (Lee & Sundar, 2013). Therefore, we hypothesize,

**Hypothesis 6:** Citing a credible source in a rumor-correction post increases the likelihood of sharing the correction post in Weibo.

Furthermore, the perception of credibility may differ by various source types (Gass & Seiter, 2008). Legitimate organizational sources (e.g., news organizations) are believed to have more resources to apply rigorous fact-checking procedures before publishing the content (Flanagin & Metzger, 2007). Conversely, a piece of personally shared information is deemed specific, narrow, and less representative of others’ views, thus resulting in an assessment of low credibility. Lee and Sundar (2013) similarly argue that the presence of “authority cues” (i.e., whether the source is an expert or not) influences people’s perception of message credibility. That is, “who” sends the message makes a difference in its subsequent retransmission on social media. Especially in social media, domain experts (e.g., health organization accounts for health messages), celebrities, and institutional actors usually garner more reposts than ordinary users (Yang et al., 2018; L. Zhang et al., 2014).

One major difference among organizational sources, celebrities, and ordinary users is the number of followers in a social platform. The number of followers is conceptualized as an indicator of user popularity (Garcia et al., 2017) or influence (Chua, Tee, et al., 2017), as it directly implies the audience size of that user (Cha et al., 2010). In a big data analysis on Twitter, Cha et al. (2010) found that follower size predicted user influence better than number of retweets or mentions when the aim was to identify users who gain extensive attention from one-to-one interaction.

More important, follower status is an indicator of credibility in social media environments. Margolin et al. (2018), for example, showed that users give more credit to a fact-checking message when the message is tweeted by someone they follow. On a similar note, Chua, Tee, et al. (2017) discovered that the number of followers is positively correlated with both the number of rumor retweets and the number of rumor-correction retweets on Twitter. In line with prior discussions, we further propose,

**Hypothesis 7:** The number of followers that (a) a rumor poster or (b) a correction poster maintains increases the likelihood of the post being shared in Weibo.
**Research Question 1:** Among different types of sources on Weibo, which source has the greatest impact on increasing the sharing of (a) rumors and (b) rumor-corrections?

## Method

### Data Collection

This study focuses on the COVID-19-related rumors and rumor-correction messages on Sina Weibo, the largest Chinese microblogging site. It serves as an open forum for discussions over heated topics and reached a record high of 550 million monthly active users in the first quarter of 2020 (Sina Finance, 2020). The hashtag *#NovelCoronavirus#* alone attracted 19.37 billion views and over 2.9 million discussions by mid-January 2021 (Sina Weibo, 2021).

We retrieved a total of 591 original rumor posts from the “CSDC-Rumor” dataset published by Tsinghua Natural Language Processing and Computational Social Science Lab (2020). These rumor posts were officially identified as false rumors by Weibo Community Management Center (2020) and published between January 22, 2020 and June 24, 2020.

We matched the correction messages provided in the CSDC-Rumor data set to their corresponding original rumors based on the Weibo Community Management Center’s feeds that linked one correction post to each reported case of false rumor posts. A total of 504 of the correction messages were linked to Weibo posts, while 15 had no links attached and 72 were linked to pages outside Weibo. We analyzed only Weibo posts. After additionally filtering out 13 mismatches and 97 pairs irrelevant to the COVID-19 pandemic, we retained 394 rumors and their corresponding correction messages for further analysis.

The respective shares and comments on the rumors and rumor-correction messages were thereafter collected via Weibo’s application programming interface.

### Measures

#### Dependent Variables

The dependent variables were the number of shares of rumor posts and their counterpart correction messages (see Table 1). The number of shares indicated the viral potential of the rumor and rumor-correction posts (Bene, 2017). On average, a rumor post attracted 99.9 shares on Weibo (SD=741.71,Mdn=2,range=12,965), while a correction message attracted 725.38 shares (SD=2,401.24,Mdn=66,range=19,578).

**Table 1.** Descriptive Statistics of the Continuous Variables.

	Rumor				Correction			
	*M*	*SD*	*Mdn*	Range	*M*	*SD*	*Mdn*	Range
Number of shares	99.90	741.71	2	12,965	725.38	2401.24	66	19,578
Follower size (in 10,000 users)	43.13	109.77			862.22	2189.81		
Emotion	2.87	4.37			2.98	3.63		
Rationality	9.57	17.60			13.26	10.27		
Length (in 10 words)	17.55	18.02			33.23	18.25		
Hashtag	0.67	1.10			1.45	1.10		

*Note.* Number of rumors = 393; number of rumor corrections = 394.

#### Independent Variables

Four independent variables were related to the content of rumors and rumor-correction messages (i.e., frames, rumor type, emotion, rationality), and two were source-level variables (i.e., types of source and the number of followers; see Tables 1 and 2).

**Table 2.** Descriptive Statistics of the Categorical Variables.

	Rumor		Correction	
	*N*	Percentage	*N*	Percentage
Frame				
Responsibility	96	24.4	92	23.4
Morality	166	42.2	84	21.3
Conflict	85	21.6	28	7.1
Human	186	47.3	167	42.4
Rumor type				
Wish	167	42.5		
Dread	132	33.6		
Wedge-driving	94	23.9		
Source type				
Public figures	150	38.2	8	2.0
Authority	0		221	56.1
News aggregator	92	23.4	11	2.8
Professional media	0		153	38.8
Ordinary users	151	38.4	1	.3
Others				
Citing credible sources			259	65.7
URL	53	13.5	205	52.0
Visual content	317	80.7	353	89.6
Total	393		394	

*Note.* Number of rumors = 393; number of rumor corrections = 394.

#### Frames

We coded the presence of four frames in both rumor and rumor-correction messages. The responsibility frame is defined as a way of attributing a cause or solution to an individual or group (Krippendorff’s α = .89 for rumor posts, 1 for correction messages). The morality frame refers to the way in which an issue was addressed based on moral or religious grounds (Krippendorff’s α = .80 for rumor posts, 1 for correction messages). The conflict frame emphasizes the confrontation or disagreement between different individuals or groups (Krippendorff’s α = 1 for both rumor posts and correction messages). Finally, the human interest frame brings “a human face or an emotional angle to the presentation of an event, issue, or problem” (Semetko & Valkenburg, 2000, p. 95; Krippendorff’s α = .77 for rumor posts, .81 for correction messages). Each frame was coded as 1 = *present* or 0 = *absent*.

#### Rumor Type

Rosnow et al. (1986) identify three types of rumors: wish, dread, and wedge-driving. We define wish rumors as rumors that project hope by positing positive outcomes; dread rumors as fear-invoking rumors that hint at negative outcomes; wedge-driving rumors promulgate hatred and division by instigating anger and hostility among audiences (Chua, Aricat, & Goh, 2017). Rumor type was coded as 0 = *wish*, 1 = *dread*, and 2 = *wedge-driving*, with wish as the reference group (Krippendorff’s α = .93).

#### Source Type

Building on Sommariva et al. (2018) and Karduni’s (2019) categorizations, we classified posters into five categories: professional media (i.e., verified Weibo accounts of mainstream news media), news aggregator (i.e., verified Weibo accounts that generally do not produce content of their own, but gather information from a variety of blogs and sites around the internet), public figures (including famous people known by their real names and social media personalities known by their pseudonyms), ordinary users (unverified accounts owned by ordinary people), and authority (including governmental sources and official rumor-rebuttal accounts; Krippendorff’s α = .78 for rumor posts, .85 for correction messages).

#### Citing Credible Sources

Credible sources were defined as genuine sources (Chua & Banerjee, 2018) that possess authority in the information they provide and are different from the users who posted the Weibo messages. Whether a message cited credible sources was coded as 1 = *present* and 0 = *absent*. This variable was unique to rumor-correction messages as rumors in the dataset rarely cited a source (Krippendorff’s α = 1).

#### Follower Size

This variable recorded the number of Weibo users that followed either the rumor posters or correctors in every 10,000. On average, a rumor poster had 411,312 followers (SD=109.77), while a corrector had 8,622,235 followers (SD=2,189.81).

#### Emotional and Rational Appeals

The other two independent variables, emotion and rationality, each address the emotional and logical rhetoric of rumors and correction messages. Emotion was operationalized as the frequency of affective words such as *qifen* (anger), *gan’en* (gratitude), and *shiwang* ([upset]; Huang et al., 2012) within each Weibo post. Rationality was operationalized as the frequency of cognitive words such as *lijie* (understand), *xuanze* (choose), and *zhiyi* (question) in each post. Interestingly, rumors (M=2.87,SD=4.37) tended to contain fewer affective words than rumor-correction messages did (M=2.98,SD=3.63). The same held true for cognitive words (rumors: M=9.57,SD=17.60; corrections: M=13.26,SD=10.27). Automatic coding was applied by using TextMind, a Chinese software equivalent to Linguistic Inquiry and Word Count (Pennebaker et al., 2015).

#### Control Variables

Studies have found that other message features, including text length (Berger & Milkman, 2012), the use of hashtag, URL, and visual content (Chua, Tee, et al., 2017) affect the sharing of online content. Therefore, these four attributes were controlled for in the statistical analysis. Length referred to the word count of each Weibo post in units of 10 words. On average, rumor-correction messages (M=33.23,SD=18.25) were longer than rumors (M=17.55,SD=18.02). Hashtag counted the number of hashtags embedded in each post. Correction messages (M=1.45,SD=1.10) tended to contain more hashtags than rumors did (M=0.67,SD=1.10). *URL* was coded as 1 if the post contained a hyperlink to external resources and 0 if it did not. Finally, visual content considered both image and video elements of a post. It was coded as 1 if either an image or a video appeared and 0 if neither was a part of the post.

### Data Analysis

Since the dependent variable—the number of shares—consisted of count data, which often had the problems of overdispersion, heteroscedasticity, and nonnormal conditional distributions of errors (Cameron & Trivedi, 2013), we chose negative binomial regression as the most appropriate model for this study. We conducted two regression analyses, one for rumor sharing and the other for rumor-correction sharing. For the rumor sharing model, the independent variables included the four frames, rumor type, the number of followers of the rumor poster, rumor emotion, rumor cognition, and rumor source identity.^1^ For the correction sharing model, the credible source citation was added to the same set of independent variables. The same control variables were used in both analyses.

## Results

### Rumor Type

Hypothesis 1 stated the relationship between rumor types and the sharing of rumors and rumor-corrections. The rumor type was not significant for the sharing of rumors (see Table 3). For rumor-corrections, compared with the correction of wish rumors, the correction of wedge-driving rumors was more likely to be shared (Β = 0.61, *p* < .05): Specifically, the correction of wedge-driving rumors engendered 1.84 (*e*^0.61^) times more shares than the correction of wish rumors. In contrast, the correction of dread rumors was not statistically different from the correction of wish rumors. Thus, Hypothesis 1b was supported while Hypothesis 1a was not.

**Table 3.** Negative Binomial Regression Results.

	Rumor				Correction			
	B	Exp(B)	Wald Chi-Square	*p*	B	Exp(B)	Wald Chi-Square	*p*
Rumor type								
Wedge-driving	0.82	2.26	3.24	.072	0.61	1.84	6.48	.011^*^
Dread	0.00	1.00	0.00	1.000	−0.29	0.75	3.17	.075
Wish	0^a^	1.00			0^a^	1.00		
Frame								
Responsibility	−0.89	0.41	4.13	.042^*^	1.95	7.04	62.54	.000^***^
Morality	0.43	1.54	1.40	.238	0.84	2.30	17.09	.000^***^
Conflict	−0.06	0.94	0.02	.881	−0.66	0.52	3.92	.048^*^
Human	0.39	1.48	1.70	.193	−0.55	0.58	10.98	.001^**^
Emotion	0.07	1.07	1.35	.246	−0.01	0.99	0.10	.747
Rationality	−0.03	0.97	1.35	.245	0.04	1.04	17.87	.000^***^
Citing credible sources					−0.14	0.87	0.72	.397
Follower size	0.003	1.003	7.50	.006^**^	0.0004	1.0004	168.41	.000^***^
Source type								
Public figures	1.97	7.20	27.47	.000^***^	1.62	5.06	1.79	.181
Authority					2.59	13.35	5.06	.024^*^
News aggregator	1.83	6.22	17.81	.000^***^	3.98	53.32	10.68	.001^**^
Professional media					2.53	12.50	4.75	.029^*^
Ordinary users	0^a^	1.00			0^a^	1.00		
Control variable								
Visual content	1.00	2.73	7.37	.007^**^	0.15	1.17	0.35	.554
Length	0.03	1.03	6.36	.012^*^	−0.03	0.97	20.74	.000^***^
URL	−1.12	0.33	5.29	.021^*^	−0.20	0.82	1.10	.295
Hashtag	−0.46	0.63	7.52	.006^**^	0.18	1.20	2.77	.096

*Note.* Number of rumors = 393; number of rumor corrections = 394.

a B = 0 because this category was used as the reference group.

* *p* < .05. ^**^p < .01. ^***^p < .001.

### Frames

Hypothesis 2 and Hypothesis 3 posited the positive effects of frames on the likelihood of sharing. The result showed that the responsibility frame (Hypothesis 2a) significantly decreased the likelihood of sharing of rumor messages (Β = −0.89, *p* < .05), contradictory to the hypothesized direction. It indicated that the presence of a responsibility frame decreased its sharing incidence rate by a factor 0.41 (*e*^−0.89^) more than a post without it. All other three frames, that is, morality (Hypothesis 2b), conflict (Hypothesis 2c), and human interest (Hypothesis 2d), were not significant.

Conversely, both the responsibility frame and morality frame significantly increased the likelihood of sharing rumor-correction messages, supporting Hypothesis 3a (Β = 1.95, *p* < .001) and Hypothesis 3b (Β = 0.84, *p* < .001), indicating that a rumor-correction was likely to be shared 7.04 (*e*^1.95^) times more if it employed the responsibility frame, and 2.3 (*e*^0.84^) times more if it employed the morality frame. Meanwhile, both conflict frame (Hypothesis 3c) and human interest frame (Hypothesis 3d) were significant but in the opposite direction: They were negatively associated with the sharing (conflict: Β = −0.66, *p* < .05; human interest: Β = −0.55, *p* < .01), indicating that a rumor-correction was likely to be shared 48% (1−*e*^−0.66^) less if it employed the conflict frame, while 42% (1−*e*^−0.55^) less if it employed the human interest frame.

### Emotion and Rationality

Hypothesis 4 and Hypothesis 5 hypothesized the positive effects of the presence of emotional and rational appeals on the size of message sharing. In terms of rumor sharing, the result showed that neither emotional appeal (Hypothesis 4a) nor rational appeal (Hypothesis 4b) had a significant effect on the sharing size of a rumor message.

Meanwhile, for a rumor-correction message, the frequency of cognitive words (Hypothesis 5b, Β = 0.04, *p* < .001) increased the likelihood of sharing, while the frequency of emotional words (Hypothesis 5a) was not a significant predictor. This indicated that a message would be shared 1.04 (*e*^0.04^) times more per one cognitive word. Therefore, Hypothesis 5b was supported while Hypothesis 5a was not.

### Source Characteristics

Hypothesis 6 posited a positive effect of citing a credible source on the likelihood of sharing rumor-corrections. No significant influence was found, however.

Hypothesis 7 posited a positive effect of follower size on the likelihood of sharing rumors and correction messages. While the result supported both Hypothesis 7a and Hypothesis 7b, the effects of the follower size were small for both rumor sharing (Β = 0.003, *p* < .01) and correction sharing (Β = 0.0004, *p* < .001).

Research Question 1 inquired about the relationship between the source types and their influence on the sharing of rumors and rumor-corrections. The results showed that, compared with ordinary users, rumor sources from public figures (Β = 1.97, *p* < .001) and news aggregators (Β = 1.83, *p* < .001) significantly increased the sharing size of a rumor, each engendering 7.2 (*e*^1.97^) times and 6.22 (*e*^1.83^) more reposts than one posted by an ordinary user.

For rumor-correction messages, compared with ordinary user, authority (Β = 2.59, *p* < .05), news aggregator (Β = 3.98, *p* < .01), and professional news media (Β = 2.53, *p* < .05) contributed to the sharing, each engendering 13.35 (*e*^2.59^) times, 53.32 (*e*^3.98^) times, and 12.5 (*e*^2.53^) times more reposts than an ordinary user. However, public figures as a rumor-correction source had no significant effect.

### Control Variables

Visual content positively predicted rumor sharing (Β = 1.00, *p* < .01, *e*^1^ = 2.73), which was in line with the findings from the literature (e.g., Chua, Tee, et al., 2017). Visual content, however, was not a significant predictor of sharing rumor-corrections.

Word count had a positive effect on rumor sharing (Β = 0.03, *p* < .05, *e*^0.03^ = 1.03), while having a negative effect on rumor-corrections (Β = −0.03, *p* < .001, 1−*e*^−0.03^ = 0.97). The inclusion of URL and the number of hashtags were negatively associated with the sharing size of rumors but had no statistically significant relationship with the sharing size of rumor-corrections.

## Discussion and Conclusions

The current study aims to understand what encourages the retransmission of COVID-19 rumors and rumor-correction messages on Sina Weibo, China. Drawing on studies of online social virality, we examined various message and source factors to understand how they affect the likelihood of a COVID-19 rumor (and its correction) post being shared.

Among the content characteristics that predict virality in social media, message frames have been relatively understudied. We compared the virality potential of different frames in both contexts of rumors and their counterpart correction posts. Overall, our findings suggest that framing effects were more prominent for the sharing of rumor-correction messages than the sharing of original rumors. For rumors, the only significant frame variable was the responsibility frame, yet in a negative manner. That is, the attribution of responsibility was less viral than other types of rumors. This result may or may not be specific to the Chinese context. Rumors that attributed responsibility to the government and the powerful were not normative in Chinese social media studied here, indicating the importance of the Chinese case; this pattern contrasts with social media in cases from the English-speaking countries, where critique of governments was common (Brennen et al., 2020).

Conversely, the responsibility frame increased the sharing of correction posts, about seven times more than those without it. This may not be surprising because responsibility attribution directly helps reduce uncertainty, which is the fundamental goal of verifying rumors. That said, it is noteworthy that the Weibo rumor-correction posts that drew much attention were not about the actors who were responsible for the COVID-19 crisis but about the actors who initiated the rumors in the first place or someone who violated government regulations. Identifying the seeds of rumors and maintaining social stability seemed the main goals underlying rumor debunking activities on Weibo.

The effect of the responsibility frame may also relate to the findings with regard to wedge-driving rumors. While wedge-driving rumors are known to spread frequently during a crisis, this study did not find its significance in terms of rumor sharing. To the contrary, the correction of wedge-driving rumors was more likely to be shared than other types of rumors. Wedge-driving rumors carry hostility against other members of the society or a certain social or political group; that is, responsibility attribution is often inherent in wedge-driving rumors. As such, the corrections of wedge-driving rumors may intend to guard social stability and harmony, reaffirming what Weibo and other Chinese social media platforms primarily intend to achieve through rumor-curbing and debunking efforts.

Along with the responsibility frame and wedge-driving rumor type, the presence of a morality frame also increased the sharing of a rumor-correction post, about twice more than without it. This finding is consistent with research on news diffusion in social media (e.g., Valenzuela et al., 2017). For one, a moral angle may resonate with audience value predisposition. For another, the presence of a moral frame may elicit either positive or negative emotions, which drive people into action. In addition to debunking the falsity, a rumor-correction message on Weibo often includes a call to action for stopping its spread. So long as a correction message laden with moral values resonates with users’ commonsense, it is likely to be shared more often.

Meanwhile, the conflict frame and human interest frame were *negative* predictors for the sharing of rumor-corrections. This finding echoes Valenzuela et al.’s (2017) point that the use of a conflict angle draws more audience interest but does not necessarily trigger more retweets. Even when it rightfully refutes false information, a conflict-oriented message can be viewed as a biased claim and thus less likely to be shared. Likewise, a human interest frame personalizes a story by bringing an emotional angle to it. The presence of a human interest frame had a decreasing effect possibly because personalized storytelling negates perceived objectivity and thus does a disservice to factuality claims.

Compared with the framing effects, rhetorical strategies showed limited effects in disseminating rumors and correction messages. The most counterintuitive finding was that an emotional appeal was insignificant for rumor sharing. This finding ran contrary to the conventional wisdom that holds that in a health crisis like COVID-19 people tend to share misinformation due to the heightened emotional tension. Prior studies have suggested that the anxiety aroused by a rumor message enhances its believability and transmission, in both off-line (Allport & Postman, 1947; Pezzo & Beckstead, 2006) and online contexts (Chua, Tee, et al., 2017; Kwon & Rao, 2017). These studies, however, have not considered framing effects, and it is possible that framing effects have overridden emotional effects.

When it comes to sharing correction posts, the minimal effect of an emotional appeal is in line with the previous findings (e.g., Chua et al., 2016). This is plausible in that the sharing of corrections must be motivated by a quest for facts and logic, as opposed to emotional expression. This fact-driven motivation may also explain why rational appeals were positively associated with the sharing of rumor-corrections. The rational appeals can enhance the legitimacy of the correction messages, which could encourage people to trust and engage with the message.

Moreover, the effects of source type were noteworthy. Consistent with prior findings (e.g., Chua, Tee, et al., 2017; Goh et al., 2017), the sources that the public deemed more legitimate than ordinary users were more effective in enhancing retransmission of both rumors and rumor-corrections. The role of news aggregators was prominent in the retransmission of rumor-correction messages: The likelihood of a news aggregator’s post being shared was 53 times higher than that of ordinary users, while other legitimate sources such as government authority and traditional media were only about 13 times more or so. News aggregators also played a role, along with public figures (mostly celebrities) in retransmissions of rumor posts. News aggregators on Weibo are distinguished from “bots” in Twitter in terms of legitimacy. While bots in Twitter could range from official news bots to spam bots and fake news bots, the news aggregators have verified Weibo accounts that specialize in releasing a variety of news. According to Weibo policy, news aggregators have to maintain more than 1,000 followers and more than 50,000 views in 30 days to attain their verification status. Government and professional news media do not have to follow such requirements. As such, news aggregators are influential in the first place, and the findings of this study show that news aggregators can contribute to the retransmission of both false rumors and correction messages.

It is also worth noting that, compared with news aggregators, government institutions and official rumor correctors—which have been considered the most respected and credible information sources in China—did not get as many shares as news aggregators. One explanation is that their posts mainly have the rhetoric of a top-down campaign and thus are less “buzzy” than those bottom-up social messages. Another explanation is that a highly credible source reduces rumor-related anxiety (Bordia et al., 2005) and thus encourages less sharing of related information. The size of followers was significant for retransmission of rumors and correction messages, which is consistent to some degree with previous studies (Chua, Tee, et al., 2017). The actual effect that this study found was relatively small, however, accounting for 3% of reposts per 100,000 followers for rumors and 4% reposts per 1,000,000 followers for rumor corrections.

The study is not free from limitations. Above all, more rumors have been spread during the pandemic, which our dataset failed to capture. We only counted falsified rumors that were paired with the corresponding correction messages, thus did not consider unverified rumors. Unverifiable rumors like conspiracy theories can sometimes be more persistent, divisive, and impactful (Sunstein & Vermeule, 2009). More important, as classical rumor psychology literature has suggested, rumors evolve into different narratives as they are transmitted (Allport & Postman, 1947; Shibutani, 1966). Given that we only analyzed the original rumor posts that were matched to the correction messages, this study did not take the evolutionary aspect of rumors into account. While this study treated rumors as static, it would be interesting to trace how a single message variegates into different versions of rumor stories as the transmission continues.

Despite the limitations, the current study is one of the first attempts to understand Chinese social media rumors and debunking messages using a content analytic approach. While this study suggests that COVID-19 rumors in China shared similar characteristics with rumors on other social media, like Twitter, their counterpart correction messages reveal unique aspects of what types of responsibility attribution are emphasized (e.g., blaming rumormongers as opposed to the triggers of COVID-19 crisis) and the role of authorities in the process of retransmission. This study serves as a starting point to broaden discussions about how to culturally situate rumor-debunking messages in order to better combat “infodemics” (United Nations, 2020) in the digital age.

## Note

1. Authority was not found in the rumor post data, and only one rumor post was sent by professional media. Accordingly, these two source types were not included in the regression analysis for rumor sharing (*N* = 393 for rumors posts; *N* = 394 for correction posts).
